# TO USE OR NOT USE PATIENT SHIELDING ON PREGNANT WOMEN UNDERGOING CT PULMONARY ANGIOGRAPHY: A PHANTOM STUDY

**DOI:** 10.1093/rpd/ncaa059

**Published:** 2020-05-19

**Authors:** Dino Begano, Marcus Söderberg, Anetta Bolejko

**Affiliations:** 1 Department of Medical Imaging and Physiology, Skåne University Hospital, Malmö SE-205 02, Sweden; 2 Medical Radiation Physics, Department of Translational Medicine, Lund University, Malmö SE-205 02, Sweden; 3 Radiation Physics, Department of Hematology, Oncology and Radiation Physics, Skåne University Hospital, Malmö SE-205 02, Sweden; 4 Department of Health Sciences, Lund University, Lund SE-221 00, Sweden

## Abstract

Pregnancy increases the risk of pulmonary embolism. Computed tomography pulmonary angiography (CTPA) is used for diagnosis. CT generates ionising radiation, and thus, abdominal shielding may be used. This phantom study investigated the effects of patient shielding and scan length reduction on the fetal and maternal ionising radiation dose from CTPA. The absorbed dose to the fetus was measured using thermoluminescent dosemeters. Estimated effective doses to the pregnant patient were based on the dose-length products. Shielding increased both the effective dose to the patient by 47% and the mean absorbed dose to the fetus (0.10 vs. 0.12 mGy; *p* < 0.001) compared with unshielded standard CTPA, as it affected the automatic exposure control. Shielded short CTPA marginally lowered only the mean fetal absorbed dose (0.03 vs. 0.02 mGy; *p* = 0.018). Shortening the scan reduced the fetal absorbed dose most effectively by 70% (0.10 vs. 0.03 mGy; *p* = 0.006), compared with the standard unshielded scan. Shielding modestly reduces fetal radiation dose but may compromise automatic exposure control, possibly increasing the maternal and fetal radiation dose. Shortening the scan is beneficial, assuming anatomical coverage is secured.

## INTRODUCTION

Untreated pulmonary embolism is a leading cause of maternal mortality during pregnancy^(1)^. Diagnostic imaging might verify pulmonary embolism and should not be delayed because of fetal radiation exposure^(2)^. However, to minimise risks associated with ionising radiation, diagnostic examinations should be performed using radiation doses that are as low as reasonably achievable (ALARA) and consistent with the diagnostic task^(3)^. Multiple imaging modalities are available for the evaluation of pulmonary embolism, including ultrasound (UL), lung scintigraphy (LS) and computed tomography (CT). Ultrasound does not use ionising radiation, but its findings do not exclude pulmonary embolism, and further diagnostic workup is required^(4)^. CT pulmonary angiography (CTPA) has been shown to be sufficient in the diagnostic workup for suspected pulmonary embolism, including in pregnancy, but LS might be preferable due to the lower radiation dose to the woman’s breast^(5)^. CTPA exposes the fetus to a similar or lower amount of radiation (0.03–0.66 mGy) compared with LS (0.32–0.74 mGy), depending on the CT scanner and imaging protocol^(1,6)^. Several studies have shown low fetal radiation doses^(7–9)^ in relation to the risk for negative effects on the fetus^(3)^. Nevertheless, it is important to continually optimise CT scan protocols according to ALARA principle.

In clinical practice, it might be common to use abdominal shielding for pregnant patients with the intention of protecting the fetus during CTPA^(10)^. For example, it was found that patient shielding in CTPA can reduce the fetal radiation dose by 39.7% despite the already low radiation dose of 0.0631 mGy^(11)^. Other studies have demonstrated that in a CT scan, most radiation to the fetus occurs from internal scattering of X-rays while they transit the patient’s body^(9, 12,13)^. Thus, abdominal shielding might reduce external fetal scattering^(13)^ but will probably do little to protect the fetus against internal scattered radiation.

Radiation procedures on pregnant patients are generally avoided because of the risk of genetic damage and teratogenicity in the fetus^(14)^. Women in early pregnancy (first trimester) are more sensitive to radiation than in late pregnancy (second and third trimester)^(15)^, as the highest risk of radiation-induced damage in the fetal central nervous system, such as mental retardation and intellectual deficits, occurs 8–15 weeks after conception^(14)^. However, since pulmonary embolism is a leading cause of maternal mortality during pregnancy, the diagnostic workup justifies radiation exposure to the fetus, but the need for CTPA should be proven by careful consideration of risks and benefits^(3,6)^. Studies have shown an overall higher mortality in patients with central as opposed to peripheral pulmonary embolism^(16)^. Thus, the CTPA scan may be shortened, since it has been suggested that emboli in the sub-segmental arteries may not contribute significantly to morbidity and mortality^(17)^. Furthermore, it has been demonstrated that the highest fetal dose reduction might be achieved by avoiding scanning the lower parts of the lungs^(18)^ and by reducing the scan length (*z*-axis)^(19)^. Optimising the tube current and voltage of the CTPA scan also might reduce the radiation dose to the pregnant patient and the fetus^(8,19)^.

In clinical practice, it might be common to place protective patient shields around the abdomen of the woman if the shield is not placed in the primary beam field, but this can jeopardise diagnostic performance; as well, the protective effect of shielding has recently been questioned^(9)^. In addition, if the patient shielding is positioned incorrectly, it might obscure the anatomy or create artefacts^(20)^. Whether patient shielding is used or not, it is important to weigh the diagnostic benefit of the examination against the risk of the procedure. Amongst pregnant women, there is a high level of anxiety associated with the use of radiation in diagnostic imaging^(15)^, so it is important to address patient concerns prior to the examination^(9)^ and provide evidence-based practice for the most effective procedures in fetal radiation protection.

The purpose of this study was to investigate the efficiency of patient shielding and the effect of reducing the scan length on fetal and patient radiation dose in CTPA.

## MATERIALS AND METHODS

**Figure 1:**
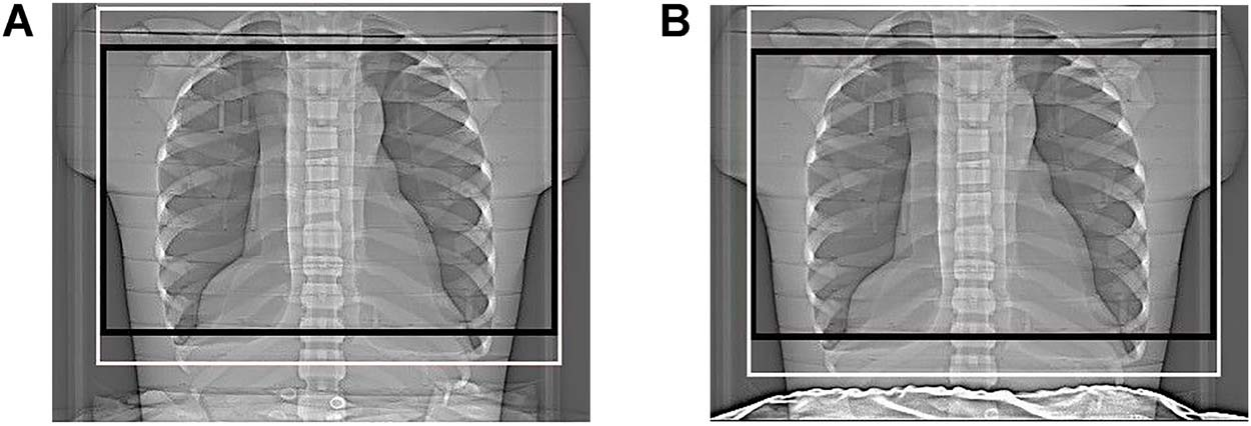
Localiser radiographs showing the CT scan area for standard and short CTPA without shielding (**A**) and with shielding (**B**). The white box corresponds to the long scanning range; the black box corresponds to the short scanning range.

In this phantom study, the effects of patient shielding around the abdomen on radiation dose from CTPA were investigated for two different scan lengths, named standard CTPA and short CTPA (Figure 1). Standard CTPA was performed according to the routine clinical protocol at the author’s site. The scan length consisted of the entire thorax, including the apex and base of the lungs. Short CTPA was set to correspond to the central part of the lungs, from the jugulum through the diaphragm. The scan range included the whole heart, while the base of the lungs was not fully included. The radiologist responsible for the CTPA protocol at the site determined the criteria for anatomical structures that were to be included.

### Phantom experiment

**Figure 2:**
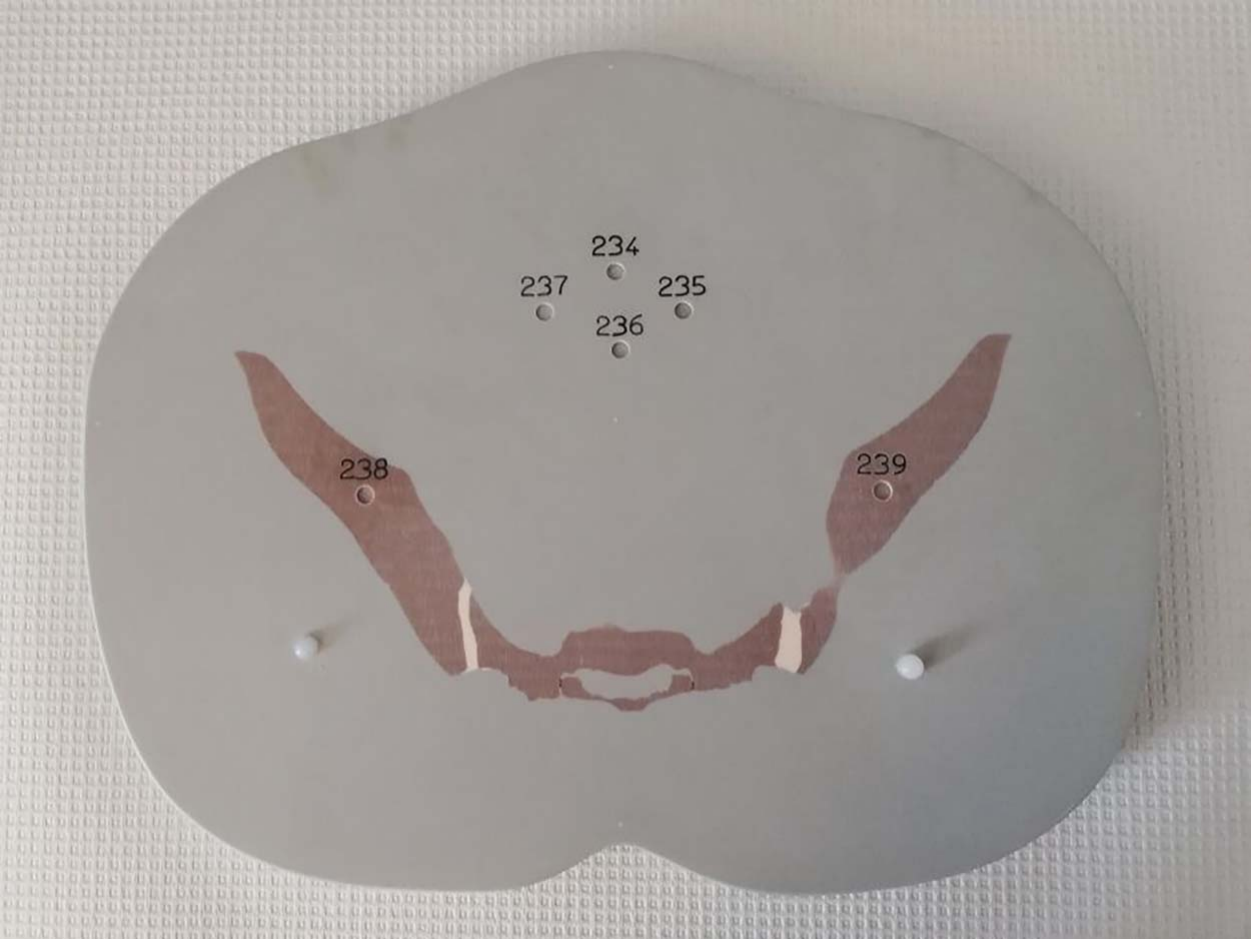
Section slab 32 of the ATOM dosimetry adult male phantom, model 701-D, with 5.5-mm wide detector holes enabling placement of dosemeters inside the phantom. TLDs were placed in positions 234–237, which represent the area of the fetal head and the woman’s uterus.

An ATOM dosimetry adult male phantom, model 701-D (CIRS, Norfolk, USA) was used^(21)^. The phantom includes spinal cord, an average bone tissue composition, and cartilage substitutes. The lung tissue substitutes mimic biological tissue. The model 701-D includes a total of 39 sectional slabs numbered 1–39 starting at the top of the head, and allows dosemeters to be placed inside the phantom through 5.5-mm wide detector holes (Figure 2).

**Figure 3:**
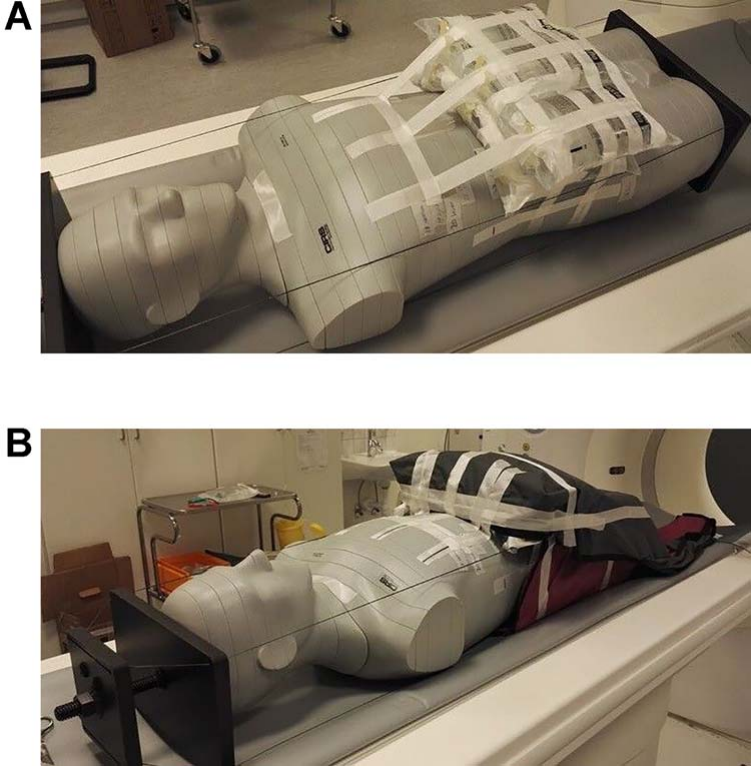
Six bags of sodium chloride solution were placed on the anterior side of the abdominal section of the ATOM dosimetry phantom to correspond to the pregnant belly (**A**) and were covered with one apron on the top of the belly and another under the abdominal part of the phantom (**B**).

In order to simulate the belly of a pregnant woman in the third trimester, six 1-L bags filled with sodium chloride solution were used. The bags were placed in front of phantom sections 23–31, corresponding to the abdominal region of the body (Figure 3), including the liver, spleen, kidneys, SI joints, and pelvis. The third trimester was chosen with the following reasoning: if a reduction of radiation dose with shielding occurs, it is expected to be most effective during this period of pregnancy.

The shielding used was two aprons, each containing 0.25 mm lead equivalent thickness (model 52333OF110, size large, Scanflex Medical AB). Before use, the aprons were checked for cracks and other ruptures. During the scanning with patient shielding, one apron was positioned on top of the manufactured belly of bags of sodium chloride solution and the other under the ATOM dosimetry phantom in order to surround the belly and the abdominal part of the phantom (Figure 3).

### CT scanning protocol

The phantom scans were performed on a Definition Flash CT (Siemens Healthineers, Forchheim, Germany). The phantom was positioned on the couch, supine, with feet towards the gantry. Each study started with an anterior-posterior localiser radiograph (120 kV, 35 mA), followed by a sequential pre-monitoring slice and three sequential monitoring slices before the actual CTPA scans were performed. All monitoring slices were placed between lumbar spine vertebrae 4 and 5 (120 kV, 20 mAs, 1 × 10). Two identical spiral scans of a range of 32 cm without and with patient shielding were performed for standard CTPA. The short CTPA scans were the same except that the scan length was 22 cm. Scanning parameters were set according to the clinical protocol at the author’s site: 120 kV reference tube voltage, 85 mAs reference tube load, pitch 1.5, rotation time 0.28 s, and detector configuration 128 × 0.6. Tube current modulation (CARE Dose 4D) and automatic tube voltage selection (CARE kV) were used as in clinical practice at the site. Shielding was included in the inferior part of the localisers but not in the region of the spiral scans (Figure 1).

### Radiation dose measurements and analyses

The radiation dose to the fetus was measured for each scan by the use of 12 thermoluminescent dosemeters (TLD-100, LiF:Mg,Ti) placed between the bags of sodium chloride (inside of the belly) and in the phantom as follows. In front of section 25, corresponding to the liver and kidneys of the patient, four TLDs were placed closed together in a quadrant inside the belly at the position corresponding to the vicinity of the fetal feet. Four other TLDs were placed in the same pattern inside the belly in front of section 29, corresponding to the crista iliaca of the patient and the location of the fetal body. In addition, four TLDs were placed in section 32 of the phantom, corresponding to the area occupied by the fetal head and the mother’s uterus (Figure 2).

The following hypotheses were investigated:

(1) Patient shielding in both standard and short CTPA is expected to decrease the fetal radiation dose.
(2) Shortening the scan length in CTPA is expected to decrease the patient and fetal radiation dose and is more effective in decreasing the fetal radiation dose than the use of patient shielding.

Each scan in every experiment was repeated five times to increase the reliability of the TLD measurements. The observed values were divided by 5 prior to analyses. The mean fetal radiation dose was calculated for each experiment based on the 12 measurement points. The mean radiation dose to the fetal head, body and feet, respectively, was calculated based on four measurement points corresponding to each region. All measured doses were corrected for background radiation based on 12 TLD measurements. A Harshaw 5500 TLD reader was used.

In addition, radiation dose calculations were performed using VirtualDose CT (Virtual Phantom Inc, Albany, USA) to verify the TLD measurements. The mean fetal dose was calculated for standard and short CTPA for virtual 9-months pregnant women. The same exposure settings as in phantom measurements were used, except that the tube load was fixed. Tube current modulation was not available in the software. The calculations were performed for the scans without patient shielding.

For each spiral scan, the volume CT dose index (CTDI_vol_; mGy) and the total dose-length product (DLP; mGy⋅cm) for each experiment were registered. An estimation of effective dose to the mother was calculated by multiplying DLP by a conversation coefficient of 0.0204 mSv per mGy⋅cm^(22)^.

Student’s paired-samples *t*-test was used to verify whether the differences in fetal radiation dose between the scans demonstrated significant outcomes. A two-tailed *p*-value <0.05 was considered to indicate significant test results. IBM SPSS version 25.0 was used for the statistical analysis.

## RESULTS

**Figure 4:**
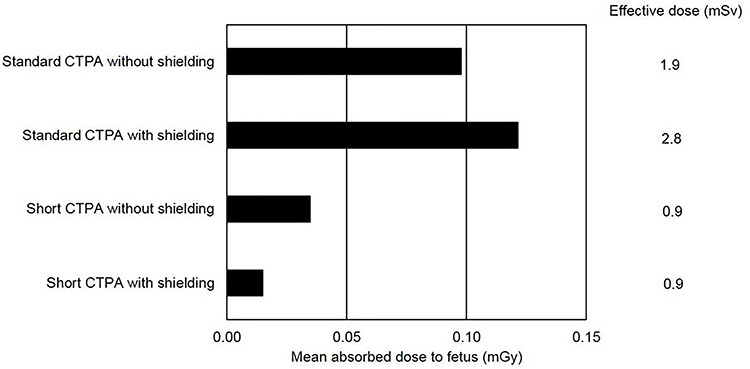
Mean absorbed dose to the fetus in milligrays (mGy) and the estimated effective dose to the pregnant woman in millisieverts (mSv).

The results showed that mean absorbed dose to the fetus was higher (0.12 mGy) when using patient shielding compared with the scan without shielding (0.10 mGy) in the standard CTPA (Table 1, Figure 4). Furthermore, the result showed a 47% (2.8 vs 1.9 mSv) higher radiation effective dose to the pregnant patient from a standard scan with shielding compared with the same scan without shielding (Figure 4). The automatic exposure control was affected by the patient shielding. Although shielding was not included in the scan area, the tube voltage was automatically increased. A voltage of 100 kV was chosen for the standard CTPA without shielding, while the tube voltage automatically increased to 120 kV in the scan with patient shielding. The mean tube current was maintained but the modulation of the tube current changed. When shielding was used, there was a relatively greater increase in tube current in the part of the scan closest to the shielding.

**Table 1:** Absorbed dose to fetus in milligrays (mGy), CT dose index (CTDI_vol_; mGy), and dose-length product (DLP; mGy·cm) and estimated effective dose to the pregnant women in millisieverts (mSv).

	Standard CTPA		Short CTPA	
	Without shielding	With shielding	Without shielding	With shielding
Fetus head (mGy)	0.020	0.032	0.004	0.005
Fetus body (mGy)	0.043	0.057	0.020	0.005
Fetus feet (mGy)	0.231	0.275	0.080	0.031
Fetus mean measured/calculated (mGy)^a^	0.10/0.08	0.12	0.03/0.03	0.02
CTDI_vol_ (mGy)	2.6	4.0	1.5	1.5
DLP (mGy·cm)	92	137	44	45
Effective dose (mSv)	1.9	2.8	0.9	0.9

^a^Calculated using VirtualDose CT.

No effect on the automatic exposure control was observed during the short CTPA scans with shielding versus without, and the radiation effective dose to the pregnant woman was 0.9 mSv in both scans (Figure 4). The mean absorbed dose to the fetus was lower (0.02 vs 0.03 mGy) when shielding was used (Table 1). The mean absorbed dose to the fetus measured with TLD corresponded well with the values that were calculated with VirtualDose CT software (Table 1).

In the standard CTPA with patient shielding, the absorbed dose to the fetus was significantly higher (*p* < 0.001) in comparison with the same scan without shielding (Table 2). The radiation absorbed dose to the fetus was significantly lower (*p* = 0.018) in short CTPA with shielding compared with the same scan without shielding (Table 2). Shortening the scan resulted in a significant (*p* = 0.006) decrease in the radiation absorbed dose to the fetus (Table 2, Figure 4).

**Table 2:** Results from the sampled Student’s *t*-test of the differences in radiation absorbed dose to the fetus at study scans.

	Mean/SD value of the difference in absorbed radiation dose to the fetus (mGy)	95% CI of the differences	*p*-value
Standard CTPA without shielding versus with shielding	−0.02/0.02	−0.03 to –0.01	<0.001
Short CTPA without shielding versus with shielding	0.02/0.03	<0.01–0.04	0.018
Standard CTPA without shielding versus short scan without shielding	0.06/0.06	0.02–0.10	0.006

## DISCUSSION

Our phantom study demonstrated that the use of patient shielding might result in increased absorbed dose to the fetus and increased effective dose to the pregnant patient. In the standard CTPA the effective dose was increased by 47% when shielding was applied, with a consequent increased radiation dose to the fetus. The short CTPA resulted in a marginally lower absorbed dose to the fetus when shielding was used, but the shielding did not alter the effective dose to the patient. Although radiation protection shielding appears to modestly reduce the fetal dose, it should be considered in relation to the introduced risk of affecting the dose modulation and increasing the radiation dose. Shortening the CT scan length had the largest effect on decreasing the radiation dose to the fetus, decreasing the mean absorbed dose by 70%.

Patient shielding is a common practice in diagnostic imaging despite growing evidence that shielding might provide only minor radiation protection and may not necessarily be beneficial^(9,23)^. Shielding may for example introduce a significant risk of increasing patient radiation dose^(9)^. As observed in our study, in the standard CTPA with shielding, the automatic exposure control was affected. We observed increased tube voltage and changed tube current modulation that consequently increased the radiation dose to the patient and therefore also to the fetus. It is important to emphasise that this was observed even though the shielding was not included in the CT spiral scan area. However, the shielding was included in the inferior part of the localiser radiograph, and this probably explains the altered X-ray output during the standard CTPA. This effect was not observed in the short CTPA, even though the shielding was slightly included in the inferior part of the localiser radiograph. It follows that when a highly attenuating material is close enough to the CT scan area, the tube output can be automatically increased. It seems that the system increases the tube output in order to penetrate the expected patient shielding, which has a higher attenuation value than the patient body, even when the shielding is not included in the CTPA scanned area but close enough to it. The automatic exposure control is supposed to calculate attenuation by the anatomy within the FOV of the scan and deliver an image with preselected quality^(9)^. Consequently, a potential dose reduction aimed at shielding the fetus introduces a risk of increasing the patient dose^(9)^, which accordingly increases the radiation dose to the fetus. It has been previously emphasised that when a lead shield is positioned incorrectly, so as to enter the imaging FOV, it might cover the area of interest and introduce artefacts^(20,23)^, compromising the diagnostic value of an image. Artefacts and obscured anatomy usually result in a repeated examination, which not only increases the radiation dose to the patient and the fetus, but also requires an additional injection of the iodine contrast agent that is used in CTPA. Iodine contrast agent can induce adverse renal reactions, and repeated injections should be carefully considered and preferably avoided, in particular amongst pregnant patients^(24,25)^. Accordingly, the use of patient shielding in pregnant patients should be questioned.

Previous studies have shown good dose reduction at shortened scan lengths, corresponding well with our results^(18,19)^. It follows that the lung bases might be excluded when performing CTPA on pregnant women^(5)^, limiting the scan length to the pulmonary arteries and branches^(26)^. Though it might be preferable to choose the standard scan as a safer solution in terms of anatomical coverage, the scan length should always be adjusted to the anatomy of interest. Uncertainty can be prevented by evidence-based practice regarding fetal radiation protection procedures.

Radiation in diagnostic imaging can provoke feelings of anxiety in pregnant women, and it is important to counsel these women prior to the examination^(15)^. It can be beneficial to address patient concerns and describe the examination to the patient^(9)^ and also to comfort the patient by explaining the benefit of the examination in relation to the potential risk of the radiation dose to the fetus^(15)^. It is also important to provide evidence-based information about the pros and cons of patient shielding and thus enable the patient to ask questions and express concerns regarding the procedure and perhaps reduce her anxiety.

The CT scans in this study were performed using automatic exposure control (CARE Dose 4D and CARE kV). This is recommended because customising the X-ray output according to the size and anatomy of the patient can contribute to a significant dose reduction^(27)^. This is the current practice at the authors’ clinic and was therefore used in the CTPA experiments in this study. Since the automatic exposure control was affected by shielding in the standard scan, the dose reduction potential of shielding would be different if fixed parameters have been used, but our aim was to follow our routine clinical guidelines. The disadvantage of using fixed parameters is that every exam gets the same radiation output regardless of patient size and attenuation and cause variation in image quality between patients and within a single scan series^(27)^.

The study was conducted on a phantom, which can be considered a limitation. But it would be unreasonable and un-ethical to conduct an experimental study on pregnant women, due to the negative effects on biological tissue of ionising radiation^(2,7,9,15)^ and the potential anxiety the radiation exposure might generate. Furthermore, placing TLD dosemeters in a patient is not possible, and therefore, the phantom is preferred. A male phantom was used that may be considered a limitation, but we had chosen to use it because its body mass may be comparable to that of a pregnant woman.

Each scan in every experiment was repeated five times to increase the reliability of the TLD measurements. Repeated measurements also controlled for the potential fluctuation of measurements as the start positon of the X-ray tube rotation is random and the TLDs were placed out of centre.

The placement of the patient shielding (Figure 3) created a sharp edge in the superior part of the belly that might have provided additional radiation protection to the fetus, but our placement does not necessarily correspond to the placement of aprons on a pregnant woman. Presumably, the minor dose reduction resulted from decreasing the external radiation to the fetus. Notably, most of the dose reduction was observed in TLDs in the area corresponding to the feet of the fetus, the superior part of the belly. In clinical practice, it may not be possible to place shielding around a pregnant patient in a similar way, due to the anatomy of the pregnant woman and her belly. Consequently, the shielding would not have protected the patient, as the radiation dose to the fetus is mainly a result of internal scattering of X-rays while they transit the patient’s body^(9,12,13)^.

Calculations using VirtualDose CT were performed in order to verify the TLD measurements, and the calculated values corresponded well with our measurements. In the dose calculation software, no dose contribution from the localiser radiograph was included since it was only possible to simulate a spiral scan, but the dose contribution from the localiser radiograph is presumably negligible. The calculations were performed for a fixed tube current and tube voltage since no automatic exposure control was available in the software. All calculations were performed without shielding since it is not possible to perform simulations with shielding. Nevertheless, we stress the value of these calculations to verify our TLD measurements, and equivalent analyses have been performed in several studies^(18,28)^.

## CONCLUSION

The use of patient shielding in CTPA should be considered in light of both its minor lessening of the radiation dose to the fetus and the risk of its increasing the dose to the patient and consequently to the fetus by affecting the automatic exposure control. Shortening the scan reduces the dose to the pregnant woman and the fetus and reduces the risk of affecting dose modulation. The scan length should be adjusted according to the prerequisites of the diagnostic workup and anatomy of interest. Future research on the development of scanning protocols is needed for evidence-based practice in diagnostic imaging. A better understanding of how to effectively translate research results to the clinic is also important, as habits of patient shielding are difficult to change. Clinical experience suggests that patient knowledge of the effects of radiation protection in CT might be biassed. Further research on how to approach patient attitudes toward and expectations of radiation protection is recommended.
